# Notch Overexpression Potentiates Interferon Signaling in Glioma Cells

**DOI:** 10.3390/cimb48060547

**Published:** 2026-05-23

**Authors:** Marina Giannaki, Elena Parmigiani, Karin Burger, Verdon Taylor, Claudio Giachino

**Affiliations:** Embryology and Stem Cell Biology, Department of Biomedicine, University of Basel, Mattenstrasse 28, 4058 Basel, Switzerland; marina.giannaki@unibas.ch (M.G.); eparmigiani@fondazionetelethon.it (E.P.); karin.burger@unibas.ch (K.B.); verdon.taylor@unibas.ch (V.T.)

**Keywords:** notch signaling, interferon signaling, brain tumor, glioma

## Abstract

Interferons (IFNs) play fundamental roles in cancer immunity. We have previously shown that conditional ablation of Notch pathway genes in a mouse model of glioma results in impaired IFNγ signaling and immunosuppressive tumors. However, it remained unclear whether the interaction between the Notch and IFN signaling pathways could be leveraged to counteract immune evasion in glioma. Here, we investigated whether expression of the intrinsically active Notch intracellular domain (NICD) could enhance IFN responses in glioma cells. Using a doxycycline (Dox)-inducible system, we overexpressed (OE) NICD in U-251MG human glioma cells. NICD-OE dramatically potentiated STAT1 phosphorylation in response to stimulation with either IFNγ or IFNα. Moreover, NICD-OE induced the expression of the transcription factor IRF1, a regulator of IFN signaling responses. Notably, NICD-OE in U-251MG human glioma cells boosted the IFNγ-dependent transcription of the *CXCL9* and *CXCL10* genes, which encode cytokines that regulate T cell function. Accordingly, NICD-OE in vivo promoted cytotoxic T lymphocyte recruitment to the tumor and reduced tumor cell proliferation in a murine glioma model. Hence, we have identified a signaling network that could be exploited to enhance anti-tumor immunity in glioma subtypes.

## 1. Introduction

Gliomas are a heterogeneous group of malignant primary brain tumors characterized by high morbidity and mortality, and limited therapeutic options [1]. Glioblastoma multiforme (GBM) represents the most aggressive form of glioma, and is almost invariably lethal despite current standard and targeted therapeutic interventions. The high aggressiveness and therapeutic resistance of GBM are determined by several factors, including invasive tumor growth, intratumoral heterogeneity, and pronounced immune evasion [2,3]. Recently, there has been considerable interest in developing immunotherapeutic approaches for GBM. However, the immunosuppressive microenvironment of these tumors poses a substantial hurdle that hinders immunotherapy in most patients [4].

Interferon (IFN) signaling plays key roles in orchestrating antitumor responses. There is mounting evidence that both type I (IFNα and IFNβ) and type II (IFNγ) IFNs can protect against tumor development. For instance, IFNs have been shown to exert cytotoxic and cytostatic effects on tumor cells, enhance antigen presentation, and promote immune responses in the tumor microenvironment (TME) [5]. IFNs can induce the expression of CXCL9 and CXCL10, two cytokines involved in the recruitment and activation of T cells, through STAT1 phosphorylation and the transcription factor IRF1 [6,7,8]. However, GBMs and even lower-grade gliomas can suppress IFN signaling via epigenetic mechanisms or genetic alterations that disrupt IFN pathway components [9,10]. Consequently, therapeutic strategies aimed at delivering exogenous IFNs or strengthening endogenous IFN signaling in gliomas have been pursued [9,11,12].

Notch signaling plays a pivotal role in the development of various types of cancer, either by promoting or suppressing tumor growth [13,14]. In a mouse model of proneural GBM, conditional ablation of single or multiple critical Notch pathway genes results in impaired IFNγ signaling and more aggressive tumors, suggesting that Notch positively regulates IFNγ responses in glioma cells [15]. However, whether Notch activation can be exploited to boost IFN responses in glioma has not been addressed. Here, we show that expression of the intrinsically active Notch intracellular domain (NICD), the transcriptional activator of the Notch signaling pathway [16], in U-251MG GBM cells can potentiate IFN signaling at multiple levels. Moreover, NICD-OE in vivo promoted cytotoxic T lymphocyte recruitment and reduced tumor cell proliferation in a murine model of glioma.

## 2. Materials and Methods

### 2.1. Cell Lines and Cell Cultures

The human GBM cell line U-251MG (Cellosaurus, RRID:CVCL_0021; https://www.cellosaurus.org/CVCL_0021 (accessed on 19 May 2026); kindly provided by Prof. Gregor Hutter, University of Basel; cell identity validated by STR profiling, Microsynth), including parental and pLIX-transduced derivatives, were maintained in DMEM (PAN Biotech GmbH, Aidenbach, Germany; P04_04510) supplemented with 1% (*v*/*v*) Penicillin-Streptomycin (Gibco, Thermo Fisher Scientific, Carlsbad, CA, USA; 15070-063) and 5% (*v*/*v*) fetal bovine serum (FBS; tetracycline-free, PAN Biotech GmbH, Aiden, Germany; P30-3602). Cells were cultured at 37 °C in a humidified atmosphere containing 5% CO_2_. For subculturing, cells were detached using TrypLE™ Express Enzyme (1×), no phenol red (Gibco, Thermo Fisher Scientific, Carlsbad, CA, USA; #11528856), following the manufacturer’s instructions. Cells were used between passages 10 and 20 and regularly checked for Mycoplasma contamination.

Lentiviral particles were produced using the second-generation packaging system consisting of psPAX2 (Addgene plasmid #12260; Addgene, Cambridge, MA, USA) and pMD2.G (Addgene plasmid #12259; Addgene, Cambridge, MA, USA), together with the transfer plasmids pLIX-hN1ICD (Addgene plasmid #91897; Addgene, Cambridge, MA, USA) or pLIX-GFP (generated by replacing the hN1ICD sequence with a cDNA encoding GFP), following published protocols [17,18]. HEK293T cells were transfected with plasmid mixtures using TransFectin™ (Bio-Rad Laboratories, Hercules, CA, USA) in serum-free DMEM according to the manufacturer’s instructions. Viral supernatants were collected 48 h post-infection, filtered through a 0.45 μm membrane and concentrated using Amicon Ultra-15 centrifugal filters (100 kDa MWCO; MilliporeSigma, Burlington, MA, USA). Aliquots of the concentrated virus (resuspended in PBS) were stored at −80 °C until use.

U-251MG cells were transduced with doxycycline (Dox)-inducible lentiviral vectors encoding either human NOTCH1 intracellular domain (pLIX-hN1ICD) or GFP (pLIX-GFP). Cells were incubated with viral particles for 3 h in the presence of polybrene (4 μg/mL), with occasional gentle pipetting, and subsequently plated in fresh medium. At 24 h post-infection, the medium was replaced, and transduced cells were selected with puromycin (0.5 μg/mL) until non-transduced cells were eliminated. Successfully transduced populations were expanded and stored in medium containing 10% DMSO for long-term preservation in liquid nitrogen.

For experimental treatments, cells were plated in the presence of Dox (1 μg/mL, Sigma-Aldrich/MilliporeSigma, Burlington, MA, USA; #D5207) to induce transgene expression. Two days after plating, cultures were treated with either human IFNα (10 ng/mL, PeproTech, Cranbury, NJ, USA; #300-02AA) or human IFNγ (10 ng/mL, PeproTech, Cranbury, NJ, USA; #AF-300-02) in the continued presence of Dox. Cells were harvested at the indicated time points after IFN treatment for protein or RNA isolation, or conditioned medium was collected for ELISA.

Brightfield images of plated U-251MG cells, treated with or without Dox and/or IFNγ, were acquired using a Nikon Ti2 Cicero confocal microscope (Nikon, Tokyo, Japan). Images were captured under standard illumination and processed using Nikon NIS-Elements software (version 6.20.02).

For the crystal violet assay, cells were stained with 0.2% crystal violet in 50% methanol. Excess dye was removed by washing the plates with water, and then the plates were air-dried. The bound crystal violet dye was solubilized in a solution containing 0.1 M Tris pH 7.5, 0.2% SDS and 20% ethanol. Absorbance was measured at 590 nm (Sinergy™ 4 plate reader, BioTek Instruments, Winooski, VT, USA).

### 2.2. Protein Extraction and Western Blotting

Cells were harvested under the indicated experimental conditions and washed twice with ice-cold PBS. Proteins were extracted in modified RIPA buffer containing 50 mM Tris-HCl (pH 8.0), 150 mM NaCl, 0.5 mM MgCl_2_, 0.5 mM EDTA, 1% Triton X-100, 0.5% SDS, 20 mM NaF, 1 mM Na_3_V0_4_ and protease inhibitors. Lysates were collected by scraping, kept on ice and intermittently vortexed. Samples were sonicated (Bioruptor Next Gen; Diagenode, Seraing, Belgium) at 4 °C to shear DNA and clarified by centrifugation at 14,000 rpm for 30 min at 4 °C. Protein concentration was determined using the Pierce™ BCA Protein Assay Kit (Thermo Fisher Scientific, Carlsbad, CA, USA).

Equal amounts of protein were mixed with Laemmli loading buffer, boiled at 95 °C for 5 min and separated by SDS-PAGE. Proteins were transferred to polyvinylidene fluoride (PVDF) membranes (Immobilion-P, Millipore, Burlington, MA, USA) using a wet transfer system at 100 V for 1 h on ice. Membranes were blocked with 5% non-fat dry milk in Tris-buffered saline containing 0.1% Tween-20 (TBST) for 1 h at room temperature and then incubated overnight at 4 °C with the following primary antibodies: anti-NOTCH1 (Cell Signaling Technology, Danvers, MA, USA; #3608, RRID: AB_2153354, 1:1000), anti-phospho-STAT1 (Cell Signaling Technology, Danvers, MA, USA; #9167, RRID: AB_561284, 1:1000), anti-STAT1α (Abcam, Cambridge, UK; #ab92506, RRID: AB_2197980), anti-IFNGR1 (Proteintech, Rosemont, IL, USA; #10808-1-AP, RRID: AB_2121604, 1:600), anti-IFNAR (Proteintech, Rosemont, IL, USA; #83002-4-RR, RRID: AB_3670748, 1:5000), anti-IRF1 (Cell Signaling Technology, Danvers, MA, USA; #8478, RRID: AB_10889027, 1:1000), anti-phospho-JAK1 (Cell Signaling Technology, Danvers, MA, USA; #74129, RRID: AB_2799851, 1:1000) anti JAK1 (Cell Signaling Technology, Danvers, MA, USA; #3344, RRID: AB_2265054, 1:1000), anti-β-actin (Sigma-Aldrich/MilliporeSigma, Burlington, MA, USA; #A5316, RRID: AB_476743, 1:5000) and anti-GAPDH (Cell Signaling Technology, Danvers, MA, USA; #97166, RRID: AB_2756824, 1:1000). After washing with TBST, membranes were incubated for 1 h at room temperature with HRP-conjugated secondary antibodies: donkey anti-rabbit IgG-HRP (Jackson ImmunoResearch, West Grove, PA, USA; #711-035-152, RRID: AB_10015282, 1:5000) and donkey anti-mouse IgG-HRP (Jackson ImmunoResearch, West Grove, PA, USA; #715-035-151, RRID: AB_2340771, 1:10,000). Protein bands were visualized using Amersham™ ECL reagents (Cytiva, Marlborough, MA, USA; Western Blotting Detection Reagent #RPN2106 and ECL Prime, Western Blotting Detection Reagent #RPN2232). Chemiluminescent signals were detected using either X-ray films or the Vilber FUSION FX imaging system. Images were analyzed using ImageJ (version 1.54t) (https://imagej.net/ij/) and densitometric quantification of protein bands was performed relative to β-actin or GAPDH as a loading control from the same membrane. Differences in signal ratios were assessed for statistical significance.

### 2.3. RNA Isolation and Quantitative Real-Time PCR Analysis

Total RNA was isolated using TRIzol™ Reagent (Thermo Fisher Scientific, Waltham, MA, USA) according to the manufacturer’s instructions with minor modifications. Briefly, 500 μL of TRIzol™ was added directly to each well of a 6-well plate, and lysates were homogenized by pipetting. Chloroform (100 μL, 20% of the total volume) was added, samples were mixed vigorously, incubated for 2–3 min at room temperature and centrifuged at 12,000× *g* for 30 min at 4 °C. The aqueous phase was collected, and RNA was precipitated overnight with isopropanol in the presence of 0.5 μL GlycoBlue™ (Invitrogen, Thermo Fisher Scientific, Waltham, MA, USA) at −20 °C. The RNA pellet was washed once with 75% ethanol, air-dried and resuspended in 20 μL RNase-free water. RNA concentration and purity were determined spectrophotometrically (NanoDrop™; Thermo Fisher Scientific, Waltham, MA, USA). Genomic DNA was removed by treating samples with Optizyme DNase I (Fisher BioReagents, Pittsburg, PA, USA) at 37 °C for 30 min, followed by enzyme inactivation with 50 mM EDTA at 65 °C for 10 min. Reverse transcription was performed using the SuperScript™ IV Reverse Transcriptase Kit (Invitrogen, Thermo Fisher Scientific, Waltham, MA, USA) with random hexamer primers (100 μM, Thermo Fisher Scientific, Waltham, MA, USA) according to the manufacturer’s instructions. Quantitative PCR was performed using gene-specific primers listed in Table 1 and PowerUp™ SYBR Green Master Mix (Applied Biosystems, Thermo Fisher Scientific, Waltham, MA, USA). Reactions were run in duplicate on a qTOWER3 G real-time PCR system (Analytik Jena, Jena, Germany) for 40 cycles. Relative mRNA expression of the genes of interest was normalized to β-actin (*ACTB*) mRNA expression and is represented as 2^−ΔCt^.

### 2.4. Enzyme-Linked Immunosorbent Assay (ELISA)

Secreted chemokines in conditioned media were quantified using Human CXCL9 (Abcam, Cambridge, UK; #ab21904) and Human CXCL10/IP-10 (Abcam, Cambridge, UK; #ab173194) ELISA kits according to the manufacturer’s instructions. Conditioned media from U-251MG pLIX-hN1ICD cells treated with Dox and IFNγ for 48 h were collected and stored at −20 °C until analysis. Standards were prepared according to the kit protocol and analyzed as single measurements, while all samples were assayed in duplicate. Three independent experiments were performed. A total of 50 μL of each sample or standard was added to the appropriate wells, followed by 50 μL of the antibody cocktail. Plates were incubated for 1 h at room temperature with gentle agitation, washed three times with 350 μL of 1× Wash Buffer PT, and 100 μL of TMB Development Solution was added to each well for 15 min in the dark. Reactions were stopped by the addition of 100 μL Stop Solution, and absorbance was measured at 450 nm using a Synergy Neo2 microplate reader (BioTek instruments, Winooski, VT, USA). Chemokine concentrations were calculated from the standard curve, and differences between experimental conditions were tested for statistical significance.

### 2.5. Animals

Floxed *Trp53*, *Rosa-CAG::GFP*, and *Notch2ICD* mice have been described previously [19,20,21]. Mice were maintained on a 12 h day/night cycle with adequate food and water under SPF conditions and in accordance with institutional regulations under license numbers 2689, 2538, and 2537, with approval dates of 1 March 2023, 1 March 2022, and 1 July 2024, and all experiments were approved by the ethics commission of the Kantonales Veterinäramt Basel-Stadt, Basel, Switzerland.

### 2.6. Retroviral Constructs, Retroviral Transduction and Generation of Murine Gliomas

The generation of the PDGFB-IRES-Cre retroviral vector has been described previously [15,22]. Replication-deficient retroviruses were generated from this construct by transfecting Platinum-E cells (Cell Biolabs, San Diego, CA, USA) using the CalPhos kit (Clontech, Takara Bio, Kusatsu, Shiga, Japan). Retroviral supernatants were harvested after 48 h. Viruses were purified using the Retro-X Concentrator kit (Clontech, Takara Bio, Kusatsu, Shiga, Japan) following the manufacturer’s instructions. Purified viruses were resuspended in TNE buffer, aliquoted and stored at −80 °C until use. The generation of murine gliomas was performed by injection of PDGFB-IRES-Cre retroviral particles into the anterior forebrain of postnatal day 2–3 mouse pups carrying floxed *Trp53* alleles, as described previously [15,22]. We induced PDGF^+^*Trp53*^−/−^ gliomas (control tumors) and PDGF^+^*Trp53*^−/−^ gliomas carrying a Cre-inducible allele (Notch2ICD allele) for the intrinsically active Notch2 intracellular domain (PDGF^+^*Trp53*^−/−^NICD^+^ tumors). For the analysis of tumors at early stages, brains were harvested 3 weeks after injection of the PDGFB-IRES-Cre virus. Brain tissue was processed and analyzed by immunostaining as described below.

### 2.7. Tissue Preparation and Immunofluorescence

Mice were deeply anesthetized by intraperitoneal injection of a ketamine/xylazine/acepromazine mixture (130 mg, 26 mg and 4 mg/kg body weight, respectively) and perfused transcardially with ice-cold 0.9% saline, followed by 4% paraformaldehyde (PFA) in 0.1 M phosphate buffer (PB, pH 7.4). Brains were post-fixed in 4% PFA at 4 °C overnight, washed in PB and cryoprotected in 30% sucrose in 0.1 M PB for 48 h. Samples were embedded in optimal cutting temperature (OCT) compound (Tissue-Tek, Sakura Finetek, Torrance, CA, USA) and frozen. Coronal sections (30 μm) were collected as free-floating sections using a cryostat and stored at −20 °C in antifreeze solution until use.

For immunostaining, sections were incubated overnight at 4 °C with blocking solution of 2% normal donkey serum (Jackson ImmunoResearch, West Grove, PA, USA), 0.5% Triton X-100 in phosphate-buffered saline (PBS) containing the following primary antibodies: anti-GFP (AbD Serotec, Kidlington, UK; #4745-1051, RRID: AB_619712, 1:500), anti-CD8α (Abcam, Cambridge, UK; #ab230156, 1:300), and anti-PCNA (Cell Signaling Technology, Danvers, MA, USA: #13110, RRID: AB_2636979, 1:3000). Sections were washed three times in PBS and incubated at room temperature for 1 h with the corresponding fluorescent-dye conjugated secondary antibodies (Jackson ImmunoResearch, West Grove, PA, USA) in blocking solution. When necessary, sections were counter-stained with DAPI (1 μg/mL). For PCNA detection, the antigen was recovered at 80 °C for 20 min in sodium citrate solution (10 mM, pH 6). Stained sections were mounted on glass slides (Thermo Scientific, Waltham, MA, USA), embedded in mounting medium containing diazabicyclo-octane (DABCO; Sigma-Aldrich/MilliporeSigma, Burlington, MA, USA) as an anti-fading agent, and visualized using a Zeiss Observer.Z1 microscope (Carl Zeiss Microscopy, Jena, Germany) equipped with Apotome.

### 2.8. Statistics

Data points shown in the figures represent independent biological replicates (for cell cultures) or individual tumor-bearing animals (for in vivo analyses). Marker-positive cells were quantified in three to four tissue sections from each animal. Statistical analyses and data visualization were performed in GraphPad Prism 10 (GraphPad Software, San Diego, CA, USA). Depending on the experimental design, either a two-tailed unpaired Student’s *t* test or one-way ANOVA followed by Tukey’s post-hoc test was used to assess statistical significance. When appropriate, statistical comparisons were conducted on data converted by arcsine square root transformation (for percentages of PCNA^+^ cells). Quantitative data are presented as mean ± SEM, and statistical significance was defined as *p* ≤ 0.05. The observed number of CD8^+^ T cells on sections was analyzed using a generalized negative binomial model, incorporating the number of tumor cells as an offset. This analysis was performed using the ‘glm.nb’ function from the MASS package (version 7.3-61) in R (version 4.4.1). A negative binomial model was chosen over the standard Poisson model, which is commonly applied to count data, due to the presence of overdispersion in the data. We used the Wald test statistic to evaluate whether the estimated coefficient differed significantly from zero.

## 3. Results

### 3.1. Generation and Characterization of NICD-OE Human Glioma Cells

**Figure 1:**
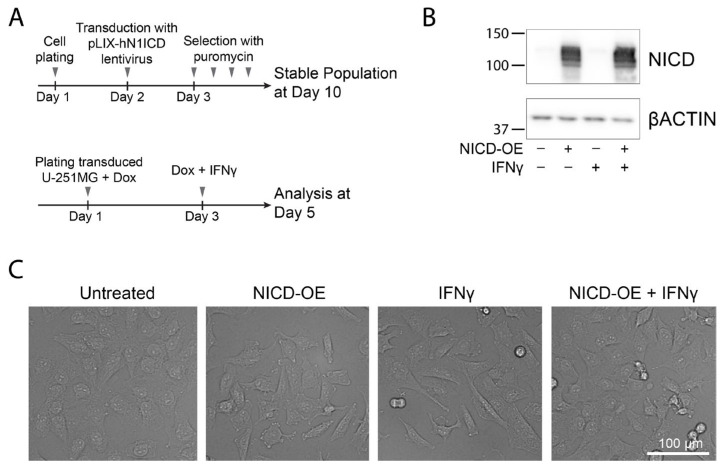
Generation of NICD-OE (Notch intracellular domain-overexpression) human glioma cells. (**A**) Scheme of the induction of NICD-OE in U-251MG glioma cells and IFNγ treatment (48 h). (**B**) Western blot analysis of NOTCH1 in U-251MG GBM cell cultures showing NICD-OE upon Dox treatment. (**C**) Representative brightfield images of U-251MG GBM cells with or without IFNγ stimulation (48 h) and Dox treatment (NICD-OE).

To investigate the impact of gain of Notch signaling on IFN signaling in glioma, we used a lentiviral-based system and generated human U-251MG glioma cells that expressed the intrinsically active NOTCH1 intracellular domain in a Dox-inducible manner (Figure 1A–C) [18]. We chose the established U-251MG cell line, which is derived from a GBM patient, because it expresses low levels of endogenous NOTCH1 protein (Figure 1B). Dox treatment robustly induced NICD expression, both in the presence or absence of IFNγ (Figure 1B). Conversely, IFNγ treatment did not dramatically affect endogenous NICD levels (Figure 1B). NICD-OE robustly induced expression of the canonical Notch target genes *HES5*, *HEY1*, and *HEY2* (Figure S1).

### 3.2. NICD-OE Potentiates IFNγ Signaling in U-251MG Cells

**Figure 2:**
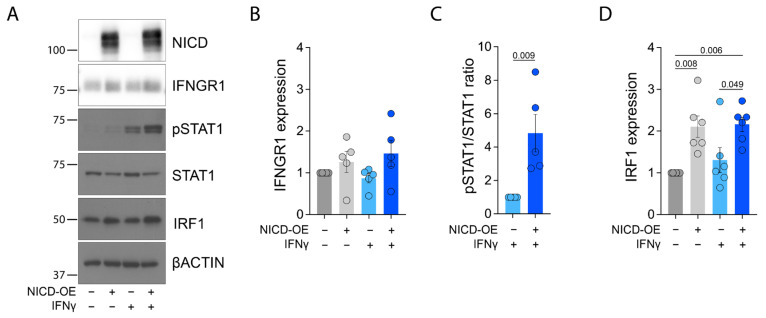
NICD-OE (Notch intracellular domain-overexpression) potentiates IFNγ signaling. (**A**) Western blot analysis of IFNγ signaling components in human U-251MG GBM cells after treatment with IFNγ (48 h) and NICD-OE. (**B**) Quantification of IFNGR1 protein expression after treatment with IFNγ and NICD-OE. Values normalized to untreated control. (**C**) Quantification of STAT1 phosphorylation (Tyr701) after treatment with IFNγ and NICD-OE. Values normalized to IFNγ treatment alone. (**D**) Quantification of IRF1 protein expression after treatment with IFNγ and NICD-OE. Values normalized to untreated control. Data represent mean ± SEM. *p* values are indicated on the graphs. One-way ANOVA with Tukey’s multiple comparisons test (**B**,**D**), Student’s *t* test (**C**); *n* = 5 (**B**,**C**) or 6 (**D**) independent experiments.

**Figure 3:**
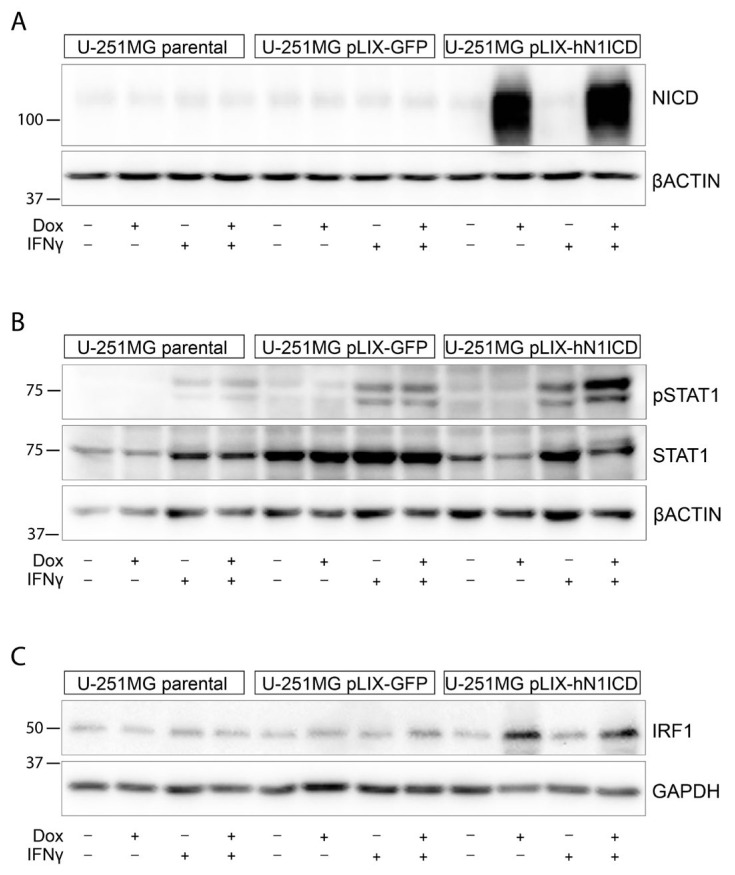
NICD-OE (Notch intracellular domain-overexpression), but not Dox treatment or GFP-OE, potentiates IFNγ signaling. (**A**) Western blot analysis of NICD in parental U-251MG cells (U-251MG parental), U-251MG cells transduced with a GFP-OE lentiviral construct (U-251MG pLIX-GFP), or U-251MG cells transduced with the NICD-OE lentiviral construct (U-251MG pLIX-hN1ICD), with or without IFNγ treatment (48 h). (**B**) Western blot analysis of STAT1 phosphorylation (Tyr701) in parental U-251MG cells, U-251MG pLIX-GFP cells, or U-251MG pLIX-hN1ICD, with or without IFNγ treatment (48 h). (**C**) Western blot analysis of IRF1 expression in parental U-251MG cells, U-251MG pLIX-GFP cells, or U-251MG pLIX-hN1ICD, with or without IFNγ treatment (48 h).

**Figure 4:**
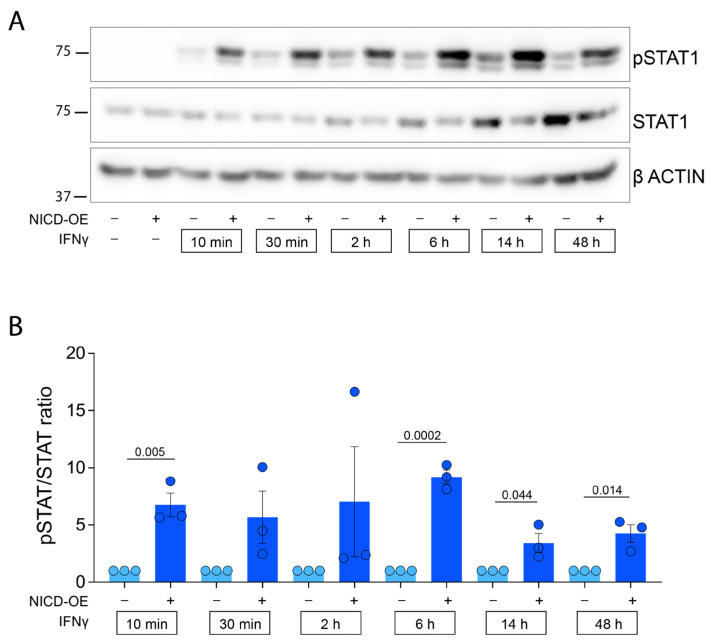
Enhanced STAT1 phosphorylation upon NICD-OE (Notch intracellular domain-overexpression) is an early response. (**A**) Western blot analysis of STAT1 phosphorylation (Tyr701) in human U-251MG GBM cells at different time points after treatment with IFNγ, with or without induction of NICD-OE. (**B**) Quantification of STAT1 phosphorylation after treatment with IFNγ and NICD-OE. Values normalized to IFNγ treatment alone. Data represent mean ± SEM. *p* values are indicated on the graphs. Student’s *t* test (**B**); *n* = 3 independent experiments (**B**).

We induced NICD-OE in U-251MG cells and analyzed IFN signaling components by Western blotting after stimulation with IFNγ. NICD-OE slightly, but not significantly, induced the expression of the IFNγ receptor 1 (IFNGR1) and its downstream effector kinase JAK1 (Figure 2A,B; Figure S2A). NICD-OE did not significantly potentiate JAK1 phosphorylation induced by IFNγ treatment (Figure S2A,B). Moreover, NICD-OE did not affect total STAT1α protein levels or induce STAT1 phosphorylation in the absence of IFNγ stimulation (Figure 2A). However, NICD-OE dramatically potentiated STAT1 phosphorylation in response to IFNγ (Figure 2A,C). In addition, NICD-OE-induced IRF1 protein and mRNA expression, both with and without IFNγ stimulation (Figure 2A,D; Figure S2C). These effects were specific to NICD-OE, as Dox treatment did not induce STAT phosphorylation or IRF1 expression in either parental, untransduced U-251MG cells, or U-251MG cells transduced with a GFP-OE lentiviral construct (Figure 3A–C). Next, we conducted a time-course experiment to determine whether NICD-OE would increase STAT1 phosphorylation even upon a brief IFNγ stimulation. We found that NICD-OE strongly induced pSTAT1 as early as 10 min after IFNγ treatment (Figure 4A,B).

### 3.3. NICD-OE Potentiates STAT1 Phosphorylation Downstream of IFNα Signaling in U-251MG Cells

**Figure 5:**
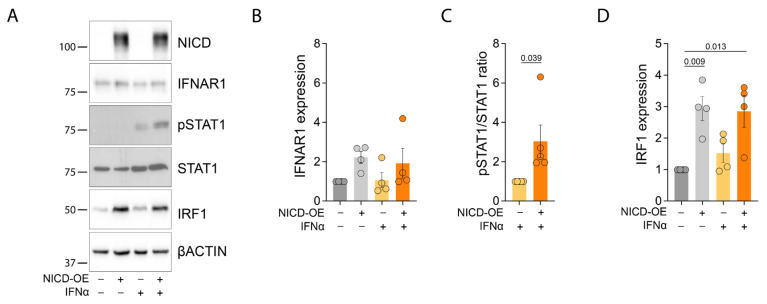
NICD-OE (Notch intracellular domain-overexpression) potentiates STAT1 phosphorylation downstream of IFNα. (**A**) Western blot analysis of IFNα signaling components in human U-251MG GBM cells after treatment with IFNα (48 h) and NICD-OE. (**B**) Quantification of IFNAR1 protein expression after treatment with IFNα and NICD-OE. Values normalized to untreated control. (**C**) Quantification of STAT1 phosphorylation (Tyr701) after treatment with IFNα and NICD-OE. Values normalized to IFNα treatment alone. (**D**) Quantification of IRF1 protein after treatment with IFNα and NICD-OE. Values normalized to untreated control. Data represent mean ± SEM. *p* values are indicated on the graphs. One-way ANOVA with Tukey’s multiple comparisons test (**B**,**D**), Student’s *t* test (**C**); *n* = 4 (**B**,**D**) or 5 (**C**) independent experiments.

Both IFNγ and IFNα play fundamental roles in cancer immunity. We addressed whether NICD-OE also potentiates IFNα signaling in glioma cells. NICD-OE did not significantly change the expression of the IFNα receptor 1 (IFNAR1) but induced IRF1 protein expression, irrespective of IFNα stimulation (Figure 5A,B,D). NICD-OE strongly potentiated STAT1 phosphorylation in the presence of IFNα (Figure 5A,C). Thus, we concluded that NICD-OE can potentiate STAT1 phosphorylation in response to stimulation with either IFNγ or IFNα.

### 3.4. NICD-OE Potentiates Cytokine Production in U-251MG Cells

**Figure 6:**
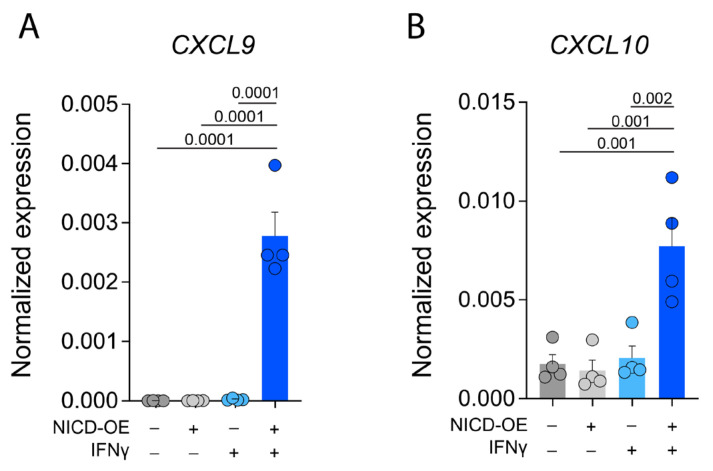
NICD-OE (Notch intracellular domain-overexpression), together with IFNγ, induces cytokine gene transcription. (**A**) qPCR analysis of *CXCL9* mRNA in human U-251MG GBM cells after treatment with IFNγ (48 h) and NICD-OE (2^−ΔCt^) (**B**) qPCR analysis of *CXCL10* mRNA in human U-251MG GBM cells after treatment with IFNγ (48 h) and NICD-OE (2^−ΔCt^). Data represent mean ± SEM. *p* values are indicated on the graphs. One-way ANOVA with Tukey’s multiple comparisons test (**A**,**B**); *n* = 4 independent experiments (**A**,**B**).

**Figure 7:**
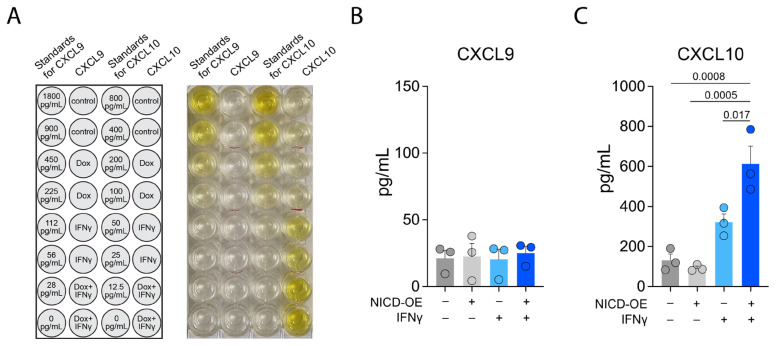
NICD-OE (Notch intracellular domain-overexpression) potentiates IFNγ-induced CXCL10 release. (**A**) Schematic diagram and representative image of a 96-well plate showing serial dilutions of standard samples and samples from the conditioned medium. (**B**) ELISA analysis of CXCL9 protein in the conditioned medium of human U-251MG GBM cells after treatment with IFNγ (48 h) and NICD-OE. (**C**) ELISA analysis of CXCL10 protein in the conditioned medium of human U-251MG GBM cells after treatment with IFNγ (48 h) and NICD-OE. Data represent mean ± SEM. *p* values are indicated on the graphs. One-way ANOVA with Tukey’s multiple comparisons test (**B**,**C**); *n* = 3 independent experiments (**B**,**C**).

IFNs, STAT1, and IRF1 are essential for the induction of the proinflammatory cytokines CXCL9 and CXCL10, which in turn can recruit T lymphocytes to the tumor [6,7]. To determine whether NICD-OE affects the expression of these cytokines in glioma, we performed qPCR analyses. NICD-OE did not induce *CXCL9* or *CXCL10* mRNA in the absence of IFNγ stimulation (Figure 6A,B; Figure S3). However, it dramatically induced *CXCL9* and *CXCL10* expression in glioma cells in concert with IFNγ as early as 14 h after IFNγ treatment (Figure 6A,B; Figure S3). We addressed whether NICD-OE altered the release of CXCL9 and CXCL10 proteins and performed ELISA on conditioned media from glioma cell cultures. CXCL9 levels were low in the medium (Figure 7A,B, Figure S4). In contrast, higher levels of CXCL10 were detected in the medium (Figure 7A,C, Figure S4). CXCL10 levels were induced by IFNγ and, strikingly, further induced in the presence of NICD-OE (Figure 7A,C, Figure S4A–D). Normalization of CXCL10 levels to cell numbers confirmed that the induction of CXCL10 by NICD-OE was not due to more cells, as cell density was slightly reduced in the NICD-OE cultures (Figure S4A,B). Hence, the Notch and IFN pathways synergize to increase the levels of proinflammatory cytokines in U-251MG glioma cells.

### 3.5. NICD-OE Alters T Cell Content of Tumors in Vivo and Inhibits Tumor Cell Proliferation in a Mouse Model of Glioma

**Figure 8:**
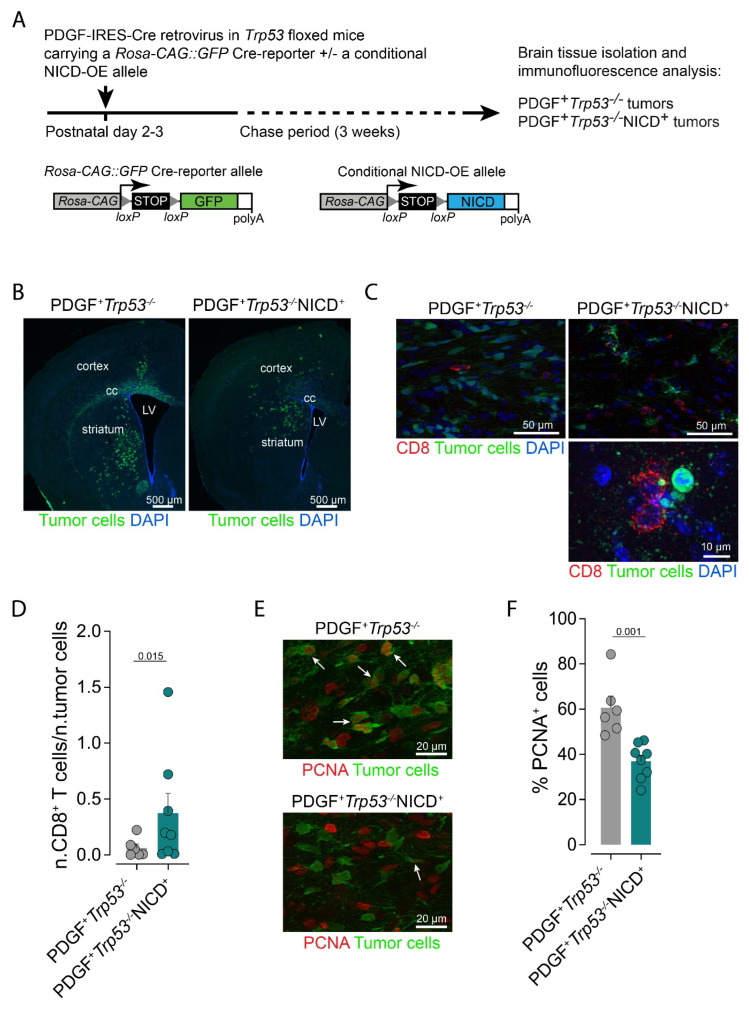
CD8^+^ T cell content and tumor cell proliferation in NICD-OE (Notch intracellular domain-overexpression) and control tumors in a mouse model of glioma in vivo. (**A**) Schematic representation of the retroviral induction of PDGF^+^*Trp53*^−/−^ and PDGF^+^*Trp53*^−/−^NICD^+^ gliomas in the mouse forebrain. The PDGFB-IRES-Cre retrovirus was stereotactically injected into the brains of early postnatal mice. The retrovirus-infected tumor cells were identified by GFP expression from the Rosa-CAG::GFP Cre-reporter locus. Cre-recombinase-induced NICD-OE from the conditional NICD-OE allele in PDGF^+^*Trp53*^−/−^NICD^+^ tumors. Early tumors (3 weeks after induction) were analyzed by immunofluorescence. (**B**) Overview images of early-stage PDGF^+^*Trp53*^−/−^ and PDGF^+^*Trp53*^−/−^NICD^+^ gliomas. Tumor cells are GFP^+^. LV, lateral ventricle; cc, corpus callosum. (**C**) Immunofluorescence images of CD8 on tissue sections from PDGF^+^*Trp53*^−/−^ and PDGF^+^*Trp53*^−/−^NICD^+^ tumors located in the corpus callosum. Note CD8^+^ cells and pyknotic GFP^+^ tumor cells that are in close proximity. (**D**) Quantification of the ratio of CD8^+^ T lymphocytes to tumor cells in PDGF^+^*Trp53*^−/−^ and PDGF^+^*Trp53*^−/−^NICD^+^ gliomas. (**E**) Images of PCNA expression on tissue sections from PDGF^+^*Trp53*^−/−^ and PDGF^+^*Trp53*^−/−^NICD^+^ tumors. PCNA^+^GFP^+^ tumor cells are indicated by arrows. (**F**) Quantification of proliferating, PCNA^+^GFP^+^ tumor cells in PDGF^+^*Trp53*^−/−^ and PDGF^+^*Trp53*^−/−^NICD^+^ gliomas. Data represent mean ± SEM. *p* values are indicated on the graphs. Significance level estimated using a negative binomial model with Wald statistics (**D**, see methods), Student’s *t* test (**F**); *n* = 6 (PDGF^+^*Trp53*^−/−^) and 8 (PDGF^+^*Trp53*^−/−^NICD^+^) independent animals (**D**,**F**).

CXCL9 and CXCL10 are chemokines involved in the recruitment and activation of T lymphocytes. To address whether NICD-OE affects T cell infiltration into tumors in vivo, we analyzed the presence of cytotoxic CD8^+^ T cells by immunofluorescence on sections from murine gliomas that overexpressed the NICD. We induced gliomas in mouse brains by retrovirus-mediated expression of PDGFB and Cre-recombinase (Cre) deletion of floxed *Trp53* alleles [15,22]. To genetically label and trace tumor cells in vivo, we used a Cre-reporter allele (Rosa-CAG::GFP) (Figure 8A). We induced PDGF^+^*Trp53*^−/−^ gliomas (control tumors) and PDGF^+^*Trp53^−^*^/−^ gliomas carrying a Cre-inducible allele for the intrinsically active Notch2 intracellular domain (PDGF^+^*Trp53*^−/−^NICD^+^ tumors) (Figure 8A,B; Figure S5). We observed CD8^+^ T cells within both PDGF^+^*Trp53^−^*^/−^ control gliomas and PDGF^+^*Trp53*^−/−^NICD^+^ gliomas (Figure 8C). Some CD8^+^ T cells were in close proximity to pyknotic, GFP+ tumor cells, suggesting cytotoxic activity (Figure 8C). Moreover, we observed an increased CD8^+^ T cell/tumor cell ratio in PDGF^+^*Trp53*^−/−^NICD^+^ gliomas, implying enhanced immune surveillance (Figure 8D). Finally, we quantified mitotically active (PCNA^+^) tumor cells (GFP^+^) and observed that they were significantly less numerous in PDGF^+^*Trp53*^−/−^NICD^+^ gliomas than PDGF^+^*Trp53*^−/−^ control tumors (Figure 8E,F).

## 4. Discussion

Aberrant Notch signaling has been linked to a variety of cancers [13,23]. In gliomas, the Notch pathway acts either as an oncogene or as a tumor suppressor, depending on the context and disease subtype [14,22,24,25,26]. Extensive crosstalk between Notch and other signaling pathways in glioma cells has been reported [27,28,29,30,31,32]. In line with these observations, we have previously shown that inhibition of Notch decreases IFNγ signaling within subtypes of gliomas [15]. Genetic deletion of *Notch1* and/or *Notch2*, or the key Notch mediator *Rbpj*, in a mouse model of proneural glioma reduces IFNγ signaling responses in the tumor cells and accelerates glioma growth [15,22]. Accordingly, treatment with the gamma secretase/Notch inhibitor DAPT reduces STAT1 phosphorylation upon IFNγ treatment in human proneural glioma cell lines [15]. Here, in a complementary gain-of-function approach, we overexpressed the intrinsically active NICD in U-251MG human glioma cells and observed enhanced responses to IFNγ or IFNα stimulation. These data confirm and extend our previous findings that Notch loss-of-function in murine proneural glioma models suppresses IFN signaling and suggest that Notch activation could be exploited to boost IFN responses in glioma subtypes. Importantly, NICD-OE reduced tumor cell proliferation and even delayed glioma growth in vivo, an effect that we and others could previously show to depend on intact, RBP-J mediated canonical Notch signaling [22,26].

We found that IFNγ- and IFNα-receptor expression did not significantly change upon increasing Notch activity in U-251MG cells. Moreover, NICD-OE did not significantly augment JAK1 phosphorylation upon IFNγ treatment. However, NICD-OE upregulated the phosphorylation of STAT1 in response to stimulation with IFNγ or IFNα, suggesting that Notch potentiates signaling downstream of the IFN receptors in U-251MG cells. In neural stem cells, the Notch effectors Hes1 and Hes5 associate with JAK2 and STAT3 to promote STAT3 phosphorylation [33]. This suggests that Notch could also facilitate the formation of JAK/STAT complexes in U-251MG glioma cells. We observed that NICD-OE induced the expression of the transcription factor IRF1, an effector of IFN signaling responses, even in the absence of IFN stimulation or detectable STAT1 phosphorylation. This finding is consistent with previous observations in macrophages, where RBP-J mediated canonical Notch signaling regulates the translation of the IRF8 protein [34]. However, we could show that NICD-OE induced both *IRF1* mRNA and IRF1 protein in U-251MG cells, suggesting that *IRF1* can be a transcriptional target of Notch in glioma subtypes. Thus, Notch can potentiate IFN and IRF1 signaling at multiple levels, either by favoring STAT activation downstream of IFN stimulation or by directly promoting the expression of IRF1.

U-251MG glioma cells with NICD-OE featured elevated cytokine transcription (*CXCL9* and *CXCL10*) and increased cytokine levels in the medium (CXCL10) downstream of IFNγ stimulation. As STAT1 and IRF1 are essential for the expression of *CXCL9* and *CXCL10*, we speculate that the enhanced STAT1 phosphorylation and increased IRF1 expression observed in the presence of NICD-OE boost the production of these cytokines [6,7]. However, we cannot rule out the possibility that Notch signaling could directly modulate *CXCL9* and *CXCL10* transcription at gene promoters, perhaps by cooperating with other signaling pathways. Consistent with this, it has been shown that RBP-J can occupy several binding sites located at intergenic enhancers between the *CXCL9* and *CXCL10* genes, suggesting that transcriptional regulation of these genes by Notch is a possibility [35]. Interestingly, murine PDGF^+^*Trp53*^−/−^ glioma cells lacking *Rbpj* have reduced mRNA expression of several cytokines, including CXCL9 and CXCL10 [15]. In the future, it would be important to determine whether NICD/RBP-J and STAT1/IRF1 synergize by binding at CXCL9/CXCL10 promoter regions or intergenic enhancers. CXCL9 and CXCL10 are chemokines involved in T cell migration and activation, and we observed that NICD-OE augments the recruitment of CD8^+^ T cells in a mouse model of glioma. Thus, Notch signaling could support the attraction of T lymphocytes by amplifying the glioma cell secretome. In addition, we have previously shown that elevated Notch signaling in proneural glioma cells could favor T cell recruitment by sustaining the expression of molecules involved in antigen processing and presentation [15].

Despite considerable challenges, including suppressed IFN signaling both in the TME and the tumor cells themselves, immunotherapy holds promise for the treatment of gliomas [4,10,15]. Neoadjuvant, but not adjuvant, anti-PD-1 immunotherapy was found to induce the downregulation of cell-cycle-related genes within tumor cells, which was attributed to increased IFNγ responsiveness, and resulted in a survival benefit for GBM patients [12]. Congruent with this, delivery of IFNα in the TME via engineered macrophages improved CAR T cell functionality, as well as endogenous T cell responses, in an immunocompetent mouse model of GBM [36]. These data strongly suggest that enhancing IFN responses in combination with immunotherapy may have synergistic effects in malignant gliomas. Hence, our findings that Notch signaling can potentiate IFN responses in U-251MG GBM cells and inhibit tumor growth in a mouse model of proneural glioma in vivo could be clinically relevant. Intriguingly, one study reported a positive correlation between elevated Notch signaling and the response to immune checkpoint blockade immunotherapy in small-cell lung cancer, although IFN responses were not directly addressed [17]. In the future, it would be interesting to test whether Notch activation can increase the efficacy of immune checkpoint blockade and/or CAR T cell therapy in mouse models of proneural GBM. Enhancing IFN signaling could be an effective therapeutic approach, particularly in proneural IDH-mutant gliomas, a disease subtype that is dependent on suppression of IFN responses for its development [9]. Noticeably, *IDH* mutations repress immunity in the tumors [37,38], and it has been suggested that *NOTCH1* inactivating mutations limit the response to inhibitors of mutant IDH [39]. Thus, it would also be important to test whether Notch activation could result in an increased response to IDH inhibitors and enhanced immunity. The Notch pathway can act both as a tumor suppressor and an oncogene in distinct forms of glioma [14]. Hence, in glioma subtypes where Notch is oncogenic, inducing Notch activity may be counter-productive. However, strategies to activate Notch signaling could be designed in glioma subtypes where Notch is tumor suppressive. Perhaps ligand-based Notch stimulation, Notch gene-delivery approaches, or the further development of recently designed soluble Notch agonists that promote T cell responses in vivo could be considered to enhance the IFN responsiveness of tumor cells and advance immunotherapeutic strategies for patients with glioma subtypes [40].

### Limitations of the Study

Analysis of more human glioma cell lines and glioma mouse models would allow us to determine whether the mechanisms that we have identified in U-251MG human glioma cells and PDGF^+^*Trp53*^−/−^ murine proneural gliomas could play a role in the development of other glioma entities. We observed reduced tumor cell proliferation and an increase in CD8^+^ T cell/tumor cell ratio in PDGF^+^*Trp53*^−/−^ gliomas upon NICD-OE. A thorough analysis of diverse immune cell components and their activation/exhaustion status in the TME would be important to determine the global effects of NICD-OE on the glioma immunophenotype and immune evasion. Although our data suggest that developing new strategies to activate Notch signaling could be beneficial in glioma subtypes where Notch acts as a tumor suppressor, potential detrimental consequences if Notch signaling is activated in glioma entities where Notch has oncogenic activities should be considered.

## Figures and Tables

**Table 1:** List of primers used for quantitative RT-PCR.

Gene	Forward Primer (5′–3′)	Reverse Primer (5′–3′)
*CXCL9*	CTGTTCCTGCATCAGCACCAAC	TGAACTCCATTCTTCAGTGTAGCA
*CXCL10*	GGTGAGAAGAGATGTCTGAATCC	GTCCATCCTTGGAAGCACTGCA
*ACTB*	CATGTACGTTGCTATCCAGGC	CTCCTTAATGTCACGCACGAT
*IRF1*	GAGGAGGTGAAAGACCAGAGCA	TAGCATCTCGGCTGGACTTCGA
*HES1*	GGAAATGACAGTGAAGCACCTCC	GAAGCGGGTCACCTCGTTCATG
*HES5*	TCCTGGAGATGGCTGTCAGCTA	CGTGGAGCGTCAGGAACTGCA
*HEY1*	TGTCTGAGCTGAGAAGGCTGGT	TTCAGGTGATCCACGGTCATCTG
*HEY2*	TGAGAAGACTTGTGCCAACTGCT	CCCTGTTGCCTGAAGCATCTTC

## Data Availability

The original contributions presented in this study are included in the article/Supplementary Materials. Further inquiries can be directed to the corresponding author(s).

## Supplementary Materials

The following supporting information can be downloaded at: https://www.mdpi.com/article/10.3390/cimb48060547/s1, Figure S1: NICD-OE induces the expression of Notch target genes; Figure S2: Analysis of JAK1 phosphorylation and *IRF1* mRNA expression. Figure S3: NICD-OE, together with IFNγ, induces *CXCL9* and *CXCL10* transcription. Figure S4: NICD-OE potentiates release of CXCL10 in the medium. Figure S5: Overview images of PDGF^+^*Trp53*^−/−^ and PDGF^+^*Trp53*^−/−^NICD^+^ gliomas. Supplementary file: Original Images of Western blots.

## Abbreviations

The following abbreviations are used in this manuscript:

CD8^+^ cell	Cytotoxic T lymphocyte
Cre	Cre-recombinase
CXCL9	C-X-C motif chemokine ligand 9
CXCL10	C-X-C motif chemokine ligand 10
Dox	Doxycycline
EDTA	Ethylenediaminetetraacetic
ELISA	Enzyme-Linked Immunosorbent Assay
GBM	Glioblastoma multiforme
GFP	Green fluorescent protein
IDH	Isocitrate Dehydrogenase
IFN	Interferon
IFNAR1	Interferon alpha/beta receptor 1
IFNGR1	Interferon gamma receptor 1
IRF	Interferon regulatory factor
JAK	Janus kinase
NICD	Notch intracellular domain
OE	Overexpression
pSTAT1	Phosphorylated signal transducer and activator of transcription 1
PB	Phosphate Buffer
PBS	Phosphate-Buffered Saline
PCNA	Proliferating Cell Nuclear Antigen
PDGFB	Platelet-derived growth factor subunit B
PFA	Paraformaldehyde
qPCR	Quantitative polymerase chain reaction
RBP-J	Recombination signal binding protein for immunoglobulin kappa J region
STAT	Signal transducer and activator of transcription protein
TME	Tumor microenvironment
