# Supplementary material for: Notch Overexpression Potentiates Interferon Signaling in Glioma Cells
Source: Curr Issues Mol Biol. 2026 May 23;48(6):547. doi: 10.3390/cimb48060547 (PMC13298569; doi:10.3390/cimb48060547)

Original images of Western blots

Figure 1

B

NOTCH1 antibody, 20µg of protein/lane,
30sec development with the Fusion FX
imaging system.
The membrane was cut before probing.
Lanes used in main Figure: 11-14.
The remaining lanes are part of a different
experiment that is not relevant to the manuscript.

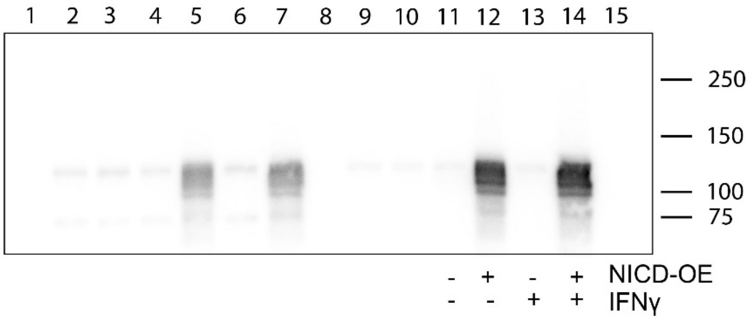

βACTIN antibody, 20µg of protein/lane,
30sec development with the Fusion FX
imaging system.
The membrane was cut before probing.
Lanes used in main Figure: 11-14.
The remaining lanes are part of a different experiment
that is not relevant to the manuscript.
βACTIN was detected at approximately 45 kDa.
The lower band represents a reprobing for GAPDH (not relevant
to the main figure).

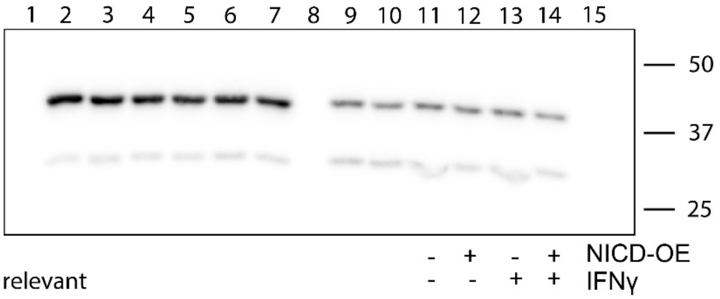

Figure 2

A

NOTCH1 antibody, 20µg of protein/lane,
30sec development with the Fusion FX
imaging system.
The membrane was cut before probing.
Lanes used in main Figure: 11-14.
The remaining lanes are not relevant
to the main figure.

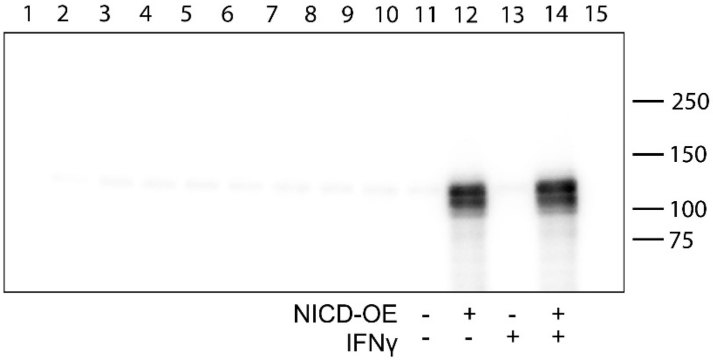

IFNGR1 antibody, 20µg of protein/lane,
30sec development with the Fusion FX imaging system.
The mature, glycosylated IFNGR1 (approximately 80-90 kDa)
was considered for densitometric analyses.

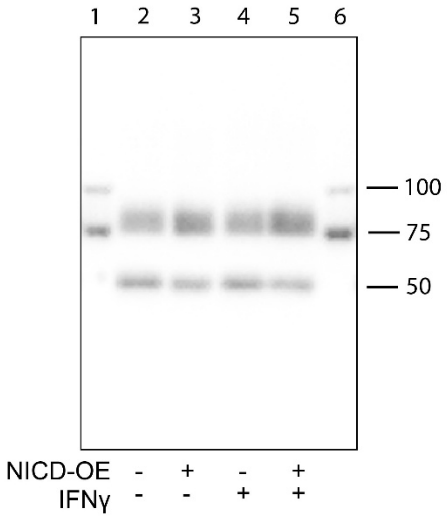

<u>pSTAT1 and STAT1α antibodies</u>, 40μg of protein/lane,
film exposure for 5min.
Lanes used in main Figure: 2-5; 7-10.
pSTAT1 and STAT1 were detected at approximately 75 kDa.
The lower band represents βACTIN (overexposed).
The membrane was cut at 50 kDa before probing.

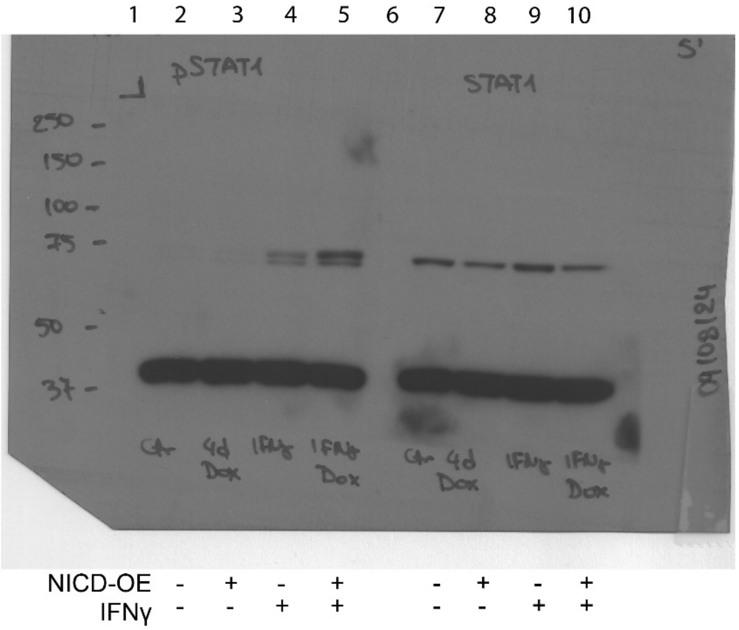

<u>IRF1 antibody</u>, 20μg of protein/lane,
film exposure for 10sec.
Lanes used in main Figure: 2-5.
The remaining lanes are part of a different experiment and are not relevant to the main figure.

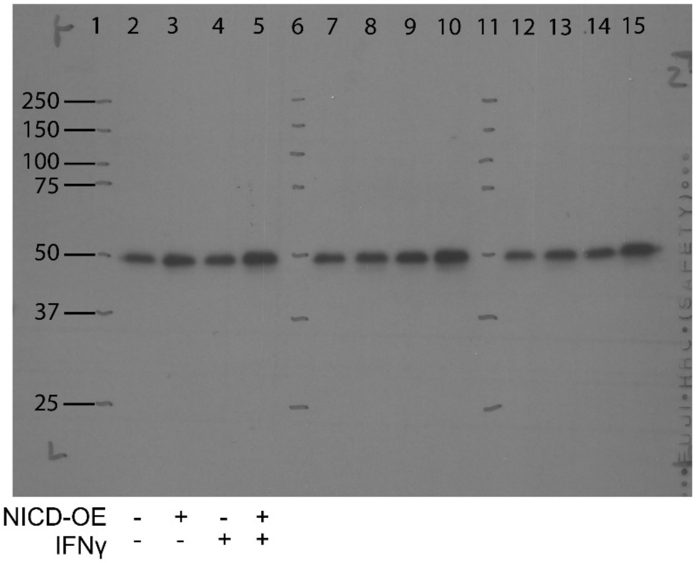

<u>βACTIN antibody</u>, 40μg of protein/lane,
film exposure for 1sec.
The membrane was cut before probing.
Lanes used in main Figure: 7-10.
The remaining lanes are part of a different experiment that is not relevant
to the manuscript.

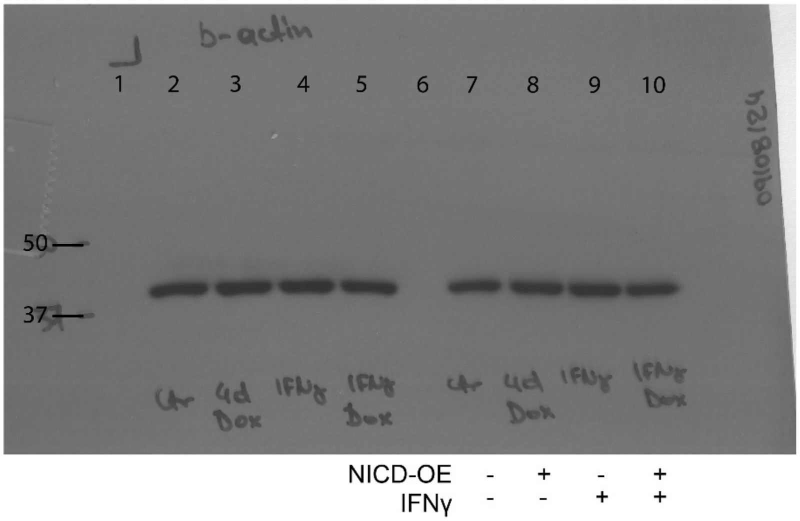

Figure 3

A

NOTCH1 antibody, 20μg of protein/lane, 30sec development with the Fusion FX imaging system.
The membrane was cut before probing.

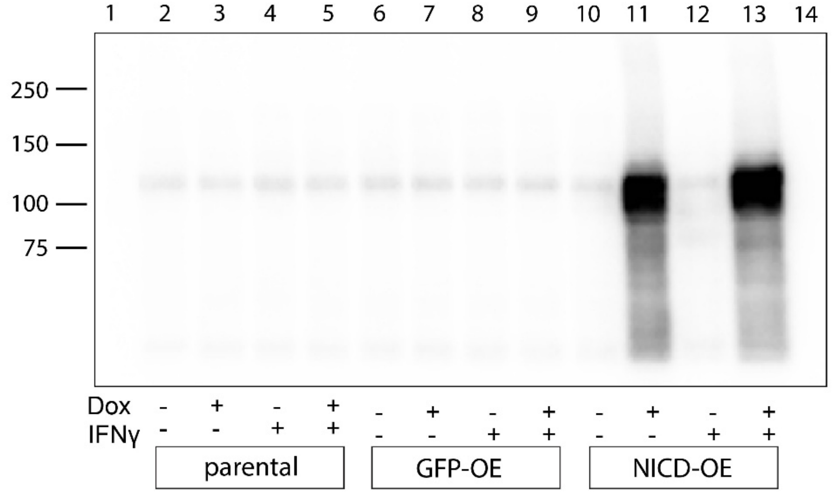

βACTIN antibody, 20μg of protein/lane, 30sec development with the Fusion FX imaging system.
The membrane was cut before probing.

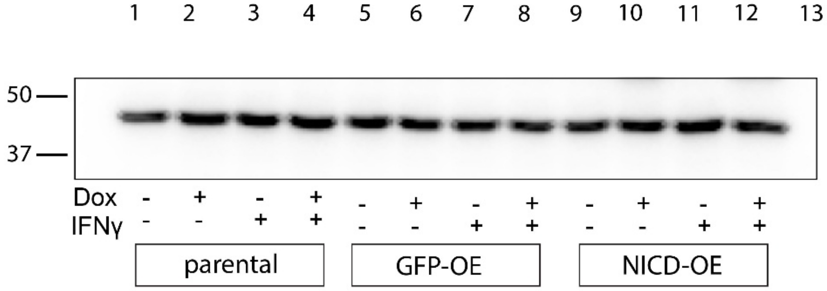

B

pSTAT1 antibody, 40μg of protein/lane, 1min development with the Fusion FX imaging system.
The membrane was cut before probing.

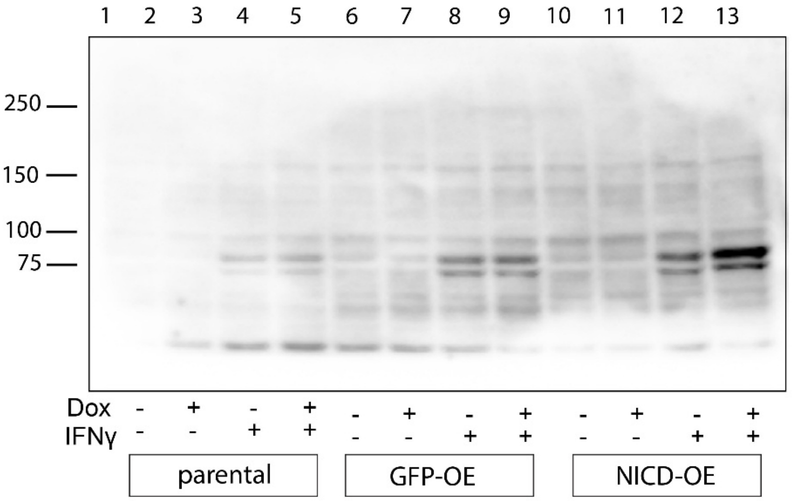

STAT1α antibody, 40μg of protein/lane, 3min development with the Fusion FX imaging system.
The membrane was cut before probing.

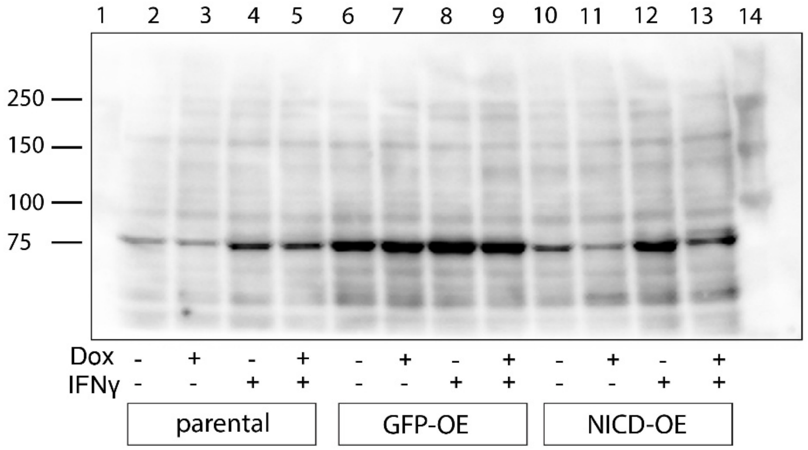

βACTIN antibody, 40µg of protein/lane, 30sec development with the Fusion FX imaging system.
The membrane was cut before probing.

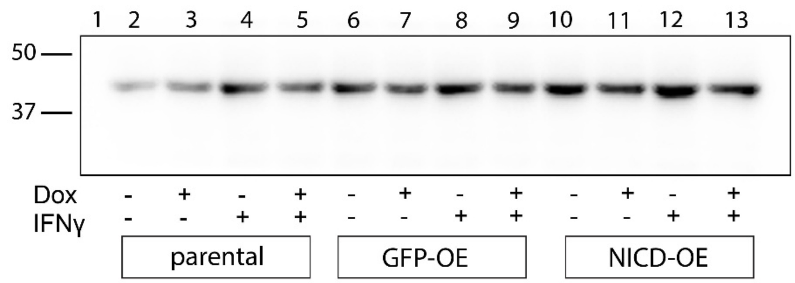

C

IRF1 antibody, 15µg of protein/lane, 6min development with the Fusion FX imaging system.
The membrane was cut before probing.

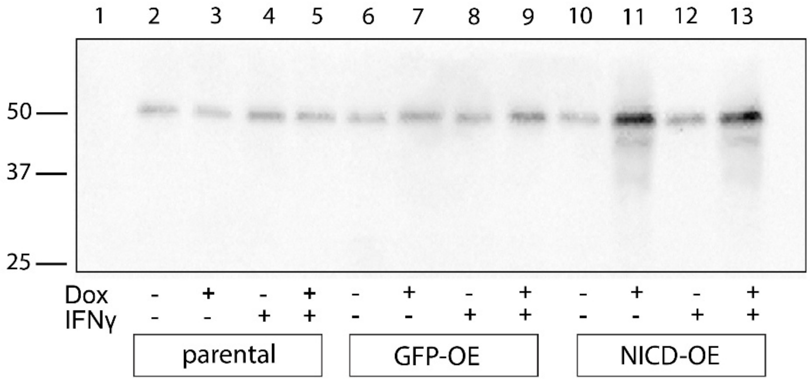

GAPDH antibody, 15µg of protein/lane, 1min development with the Fusion FX imaging system.
The membrane was cut before probing.

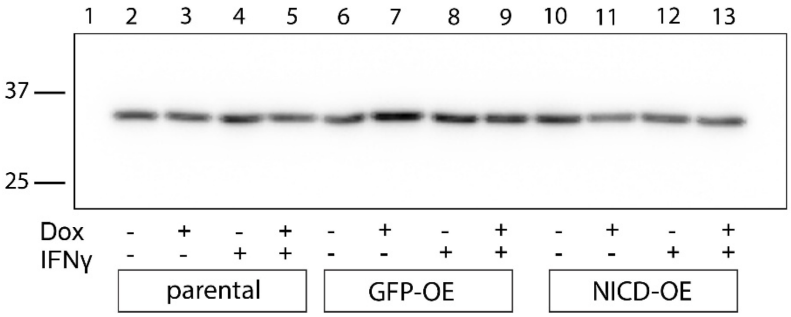

Figure 4

A

pSTAT1 antibody, 40µg of protein/lane, 30sec development with the Fusion FX imaging system.
The membrane was cut before probing.

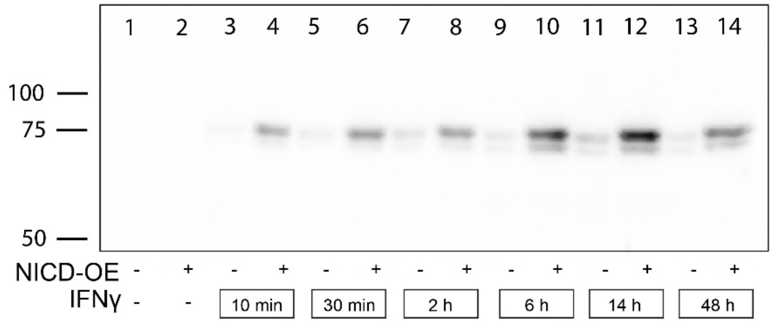

STAT1α antibody, 40µg of protein/lane, 1min development with the Fusion FX imaging system.
The membrane was cut before probing.

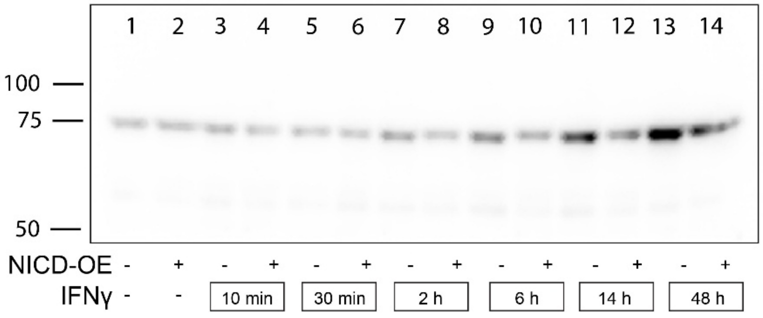

<u>βACTIN antibody</u>, 40µg of protein/lane,
30sec development with the Fusion FX
imaging system.
The membrane was cut before probing.

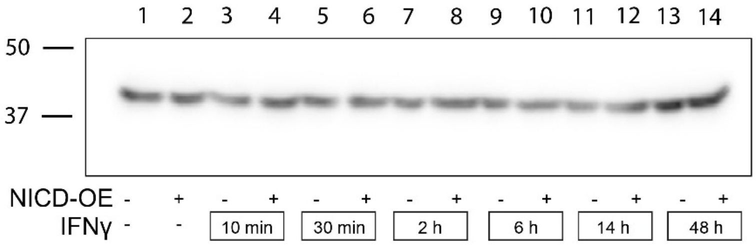

Figure 5

A

<u>NOTCH1 antibody</u>, 20µg of protein/lane,
30sec development with the Fusion FX
imaging system.
Lanes used in main Figure: 2-5.
The remaining lanes are part of a different
experiment that is not relevant to the manuscript.
NOTCH1 was detected at approximately 110 kDa.
The lower band represents a reprobing
for βACTIN (not relevant to the main figure).

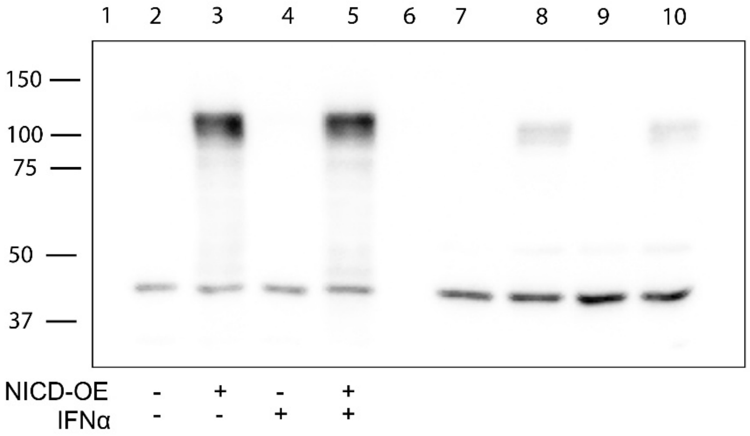

<u>IFNAR1 antibody</u>, 20µg of protein/lane,
30sec development with the Fusion FX
imaging system
The membrane was cut before probing.
Lanes used in main Figure: 2-5.
The remaining lanes are part of a different experiment
that is not relevant to the manuscript.

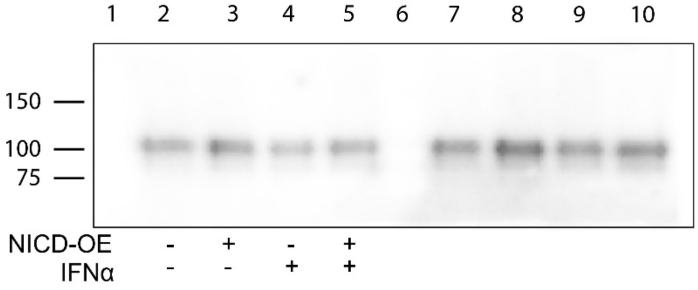

<u>pSTAT1 and STAT1α antibodies</u>, 40µg of protein/lane,
film exposure for 1min.
The membrane was cut before probing.
Lanes used in main Figure: 2-5; 7-10.

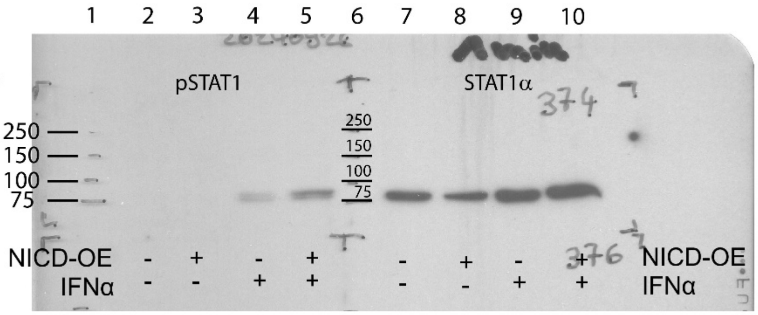

IRF1 antibody, 40μg of protein/lane,
30sec development with the Fusion FX imaging system.
The membrane was cut before probing.
Lanes used in main Figure: 2-5.
The remaining lanes are part of a different experiment that is not relevant to the manuscript.

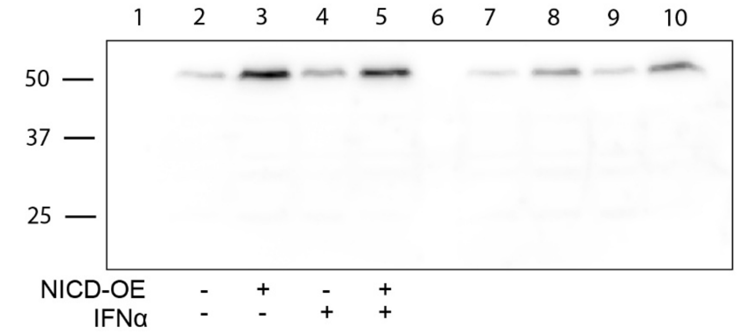

βACTIN antibody, 40μg of protein/lane,
30sec development with the Fusion FX imaging system.
The membrane was cut before probing.
Lanes used in main Figure: 2-5.
The remaining lanes are part of a different experiment that is not relevant to the manuscript.

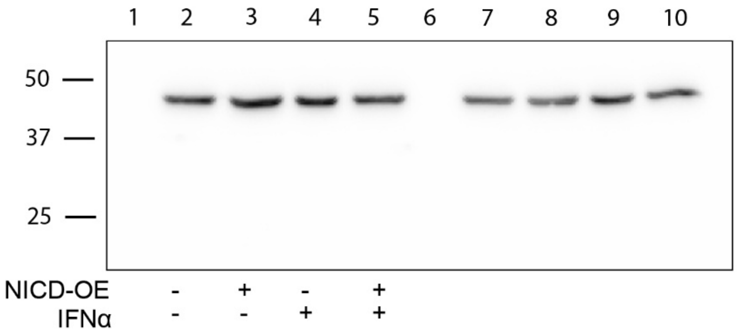

Supplementary Figure 2A

pJAK1 and JAK1 antibodies,
30μg of protein/lane,
film exposure for 20min.
The membrane was cut before probing.
Lanes used in Figure S2A: 6-9.
The remaining lanes are part of a different experiment that is not relevant to the figure.

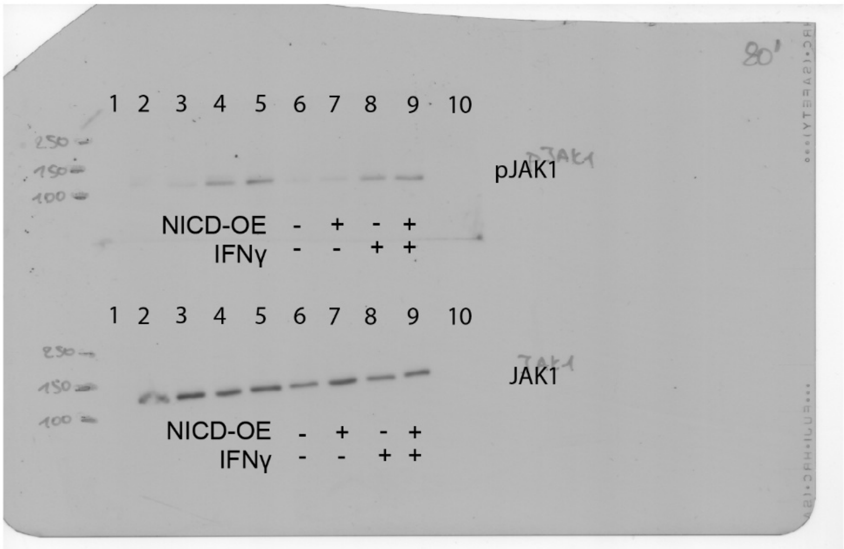

βACTIN antibody, 30μg of protein/lane,
film exposure for 5sec.
The membrane was cut before probing.
Lanes used in Figure S2A: 6-9 (lower membrane, bottom right).
The remaining lanes are part of a different experiment that is not relevant to the figure.

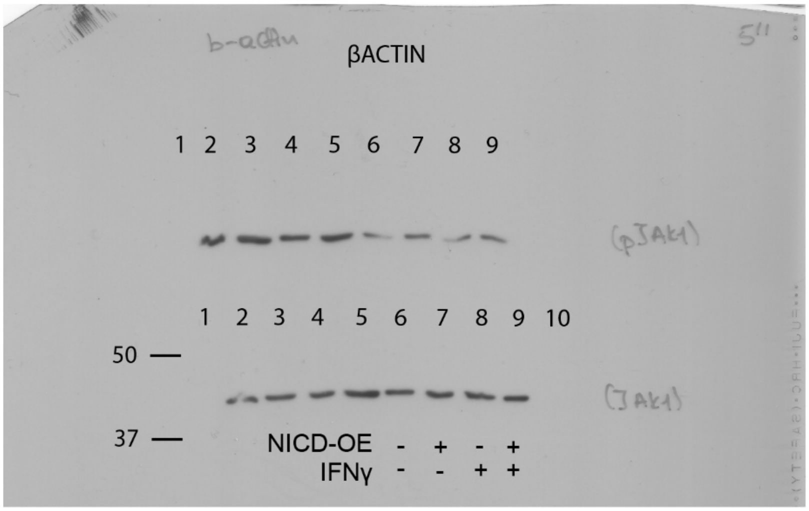

Supplement: Supplementary file 1 [file cimb-48-00547-s001.zip › Original Images of Western blots.pdf]
