# Supplementary material for: Notch Overexpression Potentiates Interferon Signaling in Glioma Cells
Source: Curr Issues Mol Biol. 2026 May 23;48(6):547. doi: 10.3390/cimb48060547 (PMC13298569; doi:10.3390/cimb48060547)
Supplement: Supplementary file 1 [file cimb-48-00547-s001.zip › Supplementary Figure Legends.pdf]

Supplementary Figure Legends

Figure S1. NICD-OE induces the expression of Notch target genes. (**A**) qPCR analysis of *HES1, HES5, HEY1,* and *HEY2* mRNAs in human U-251MG GBM cells after treatment with IFNγ (48h) and NICD-OE ($2^{-\Delta Ct}$). Data represent mean ± SEM. p values are indicated on the graphs. One-way ANOVA with Tukey's multiple comparisons test; n=3 independent experiments (A).

Figure S2. Analysis of JAK1 phosphorylation and *IRF1* mRNA expression. (**A**) Western blot analysis of pJAK1 in human U-251MG GBM cells after treatment with IFNγ (48h) and NICD-OE. (**B**) Quantification of JAK1 phosphorylation after treatment with IFNγ and NICD-OE. Values normalized to IFNγ treatment alone. (**C**) qPCR analysis of *IRF1* mRNA in human U-251MG GBM cells after treatment with IFNγ and NICD-OE ($2^{-\Delta Ct}$). Data represent mean ± SEM. p values are indicated on the graphs. Student's t test (B), One-way ANOVA with Tukey's multiple comparisons test (C); n=4 (B) or 6 (C) independent experiments.

Figure S3. NICD-OE, together with IFNγ, induces *CXCL9* and *CXCL10* transcription. (**A**) qPCR analysis of *CXCL9* mRNA in human U-251MG GBM cells at different time points after treatment with IFNγ, with or without induction of NICD-OE ($2^{-\Delta Ct}$). (**B**) qPCR analysis of *CXCL10* mRNA in human U-251MG GBM cells at different time points after treatment with IFNγ, with or without induction of NICD-OE ($2^{-\Delta Ct}$). Data represent mean ± SEM. p values are indicated on the graphs. One-way ANOVA with Tukey's multiple comparisons test (A,B); n=3 independent experiments (A,B).

Figure S4. NICD-OE potentiates release of CXCL10 in the medium. (**A**) Quantification of cell numbers with crystal violet assay (U-251MG GBM cells). Values normalized to untreated control. (**B**) ELISA analysis of CXCL10 protein in the conditioned medium of human U-251MG GBM cells after treatment with IFNγ (48h) and NICD-OE. Values (pg/mL) were normalized to cell numbers estimated by crystal violet staining. (**C**) ELISA analysis of CXCL9 protein in the conditioned medium of human U-251MG GBM cells at different time points after treatment with IFNγ, with or without induction of NICD-OE. Values normalized to untreated control. (**D**) ELISA analysis of CXCL10 protein in the conditioned medium of human U-251MG GBM cells at different time points after treatment with IFNγ, with or without induction of NICD-OE. Values normalized to untreated control. Data represent mean ± SEM. p values are indicated on the graphs. One-way ANOVA with Tukey's multiple comparisons test (A-D); n=3 independent experiments (A-D).

Figure S5. Overview images of $PDGF^{+}$*Trp53*$^{-/-}$ and $PDGF^{+}$*Trp53*$^{-/-}$$NICD^{+}$ gliomas. (**A**) Low magnification, overview images of early-stage tumors from coronal sections of the mouse brain. Tumor cells are GFP^{+}. Sections are counterstained with DAPI.
