# Supplementary figures and images for: Notch Overexpression Potentiates Interferon Signaling in Glioma Cells
Source: Curr Issues Mol Biol. 2026 May 23;48(6):547. doi: 10.3390/cimb48060547 (PMC13298569; doi:10.3390/cimb48060547)

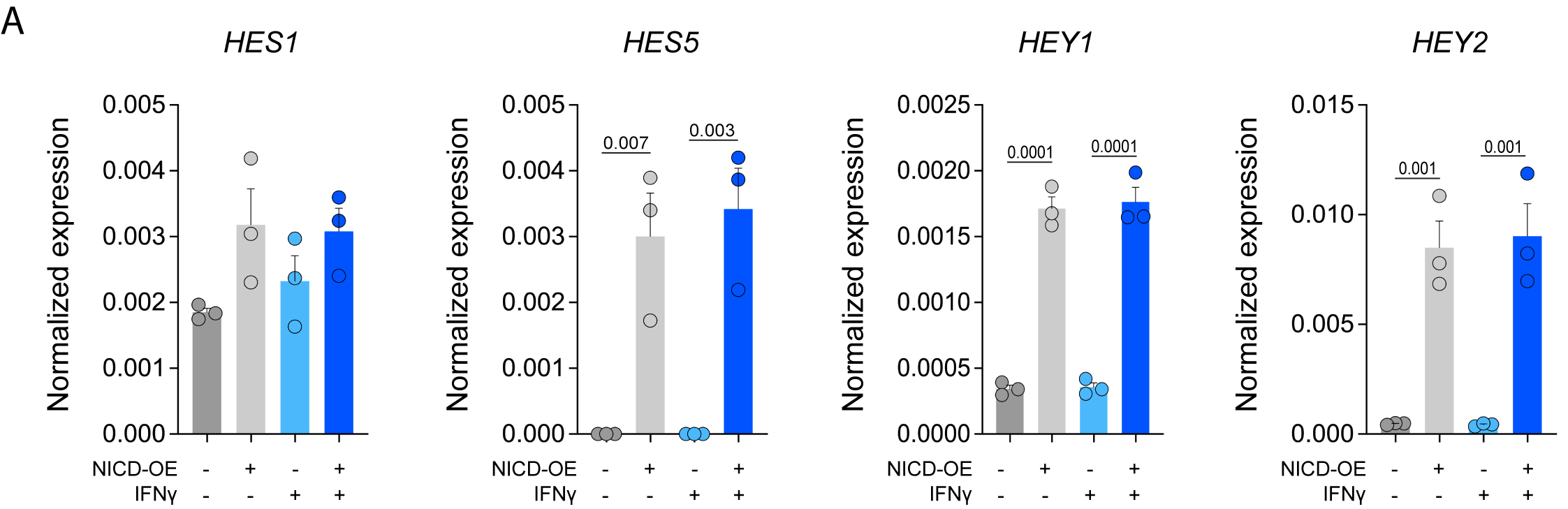

Supplement: Supplementary file 1 [file cimb-48-00547-s001.zip › Figure S1.tif]

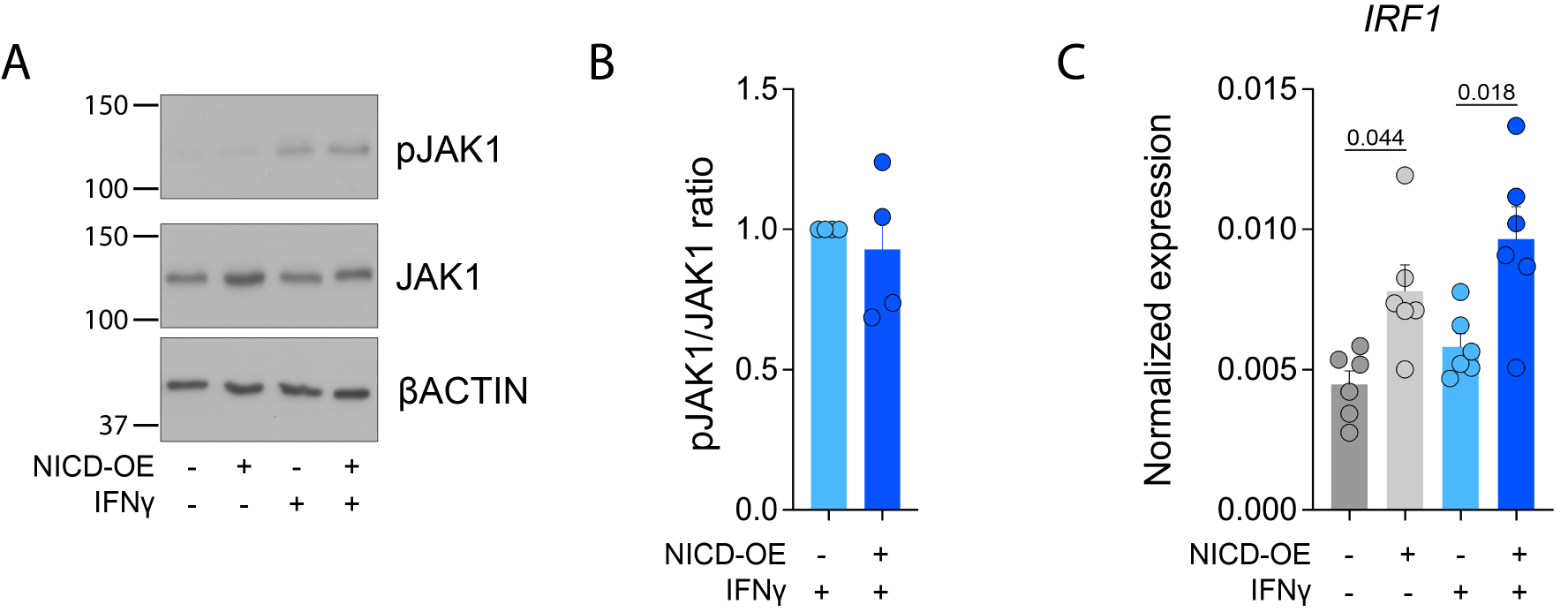

Supplement: Supplementary file 1 [file cimb-48-00547-s001.zip › Figure S2.tif]

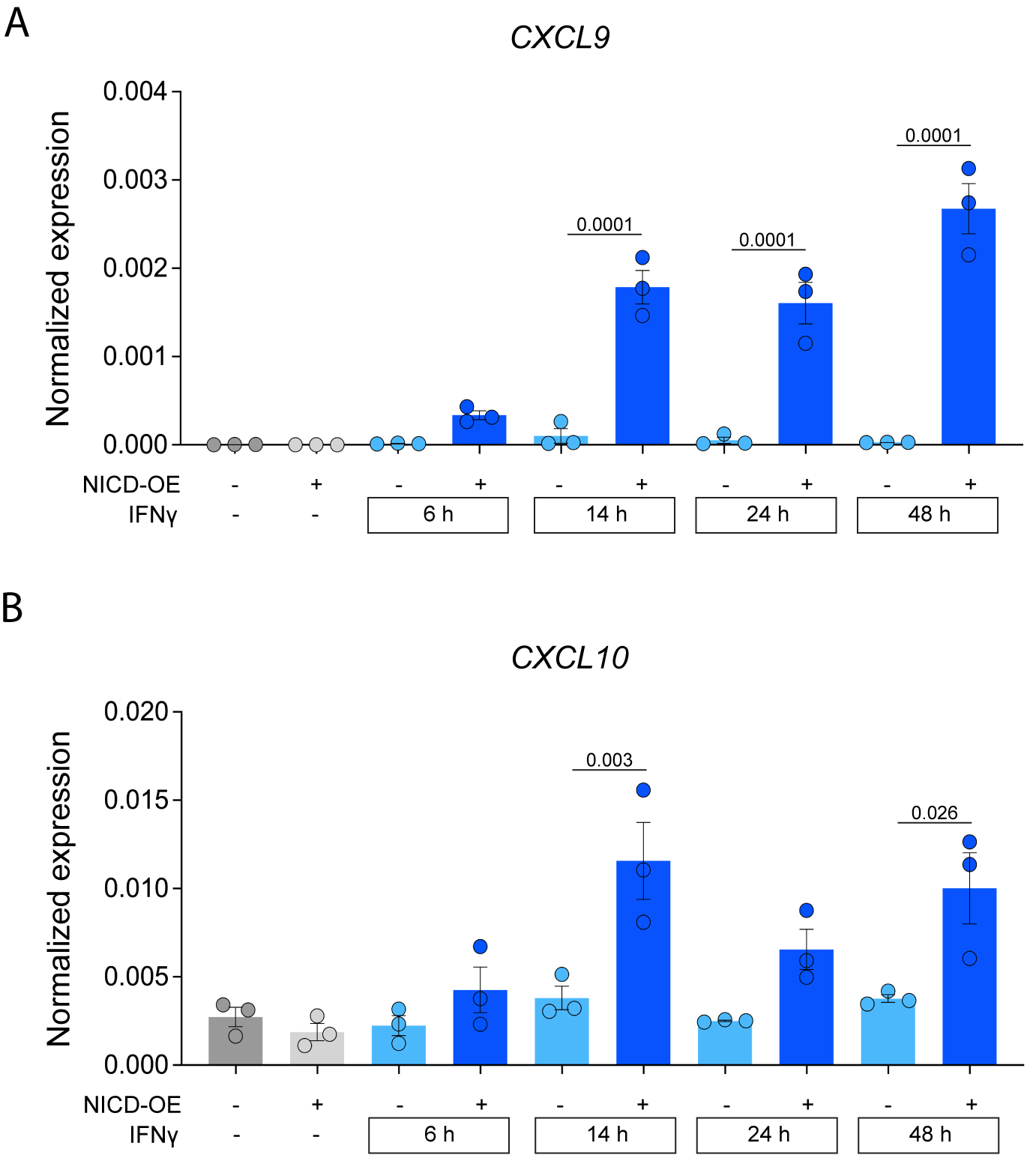

Supplement: Supplementary file 1 [file cimb-48-00547-s001.zip › Figure S3.tif]

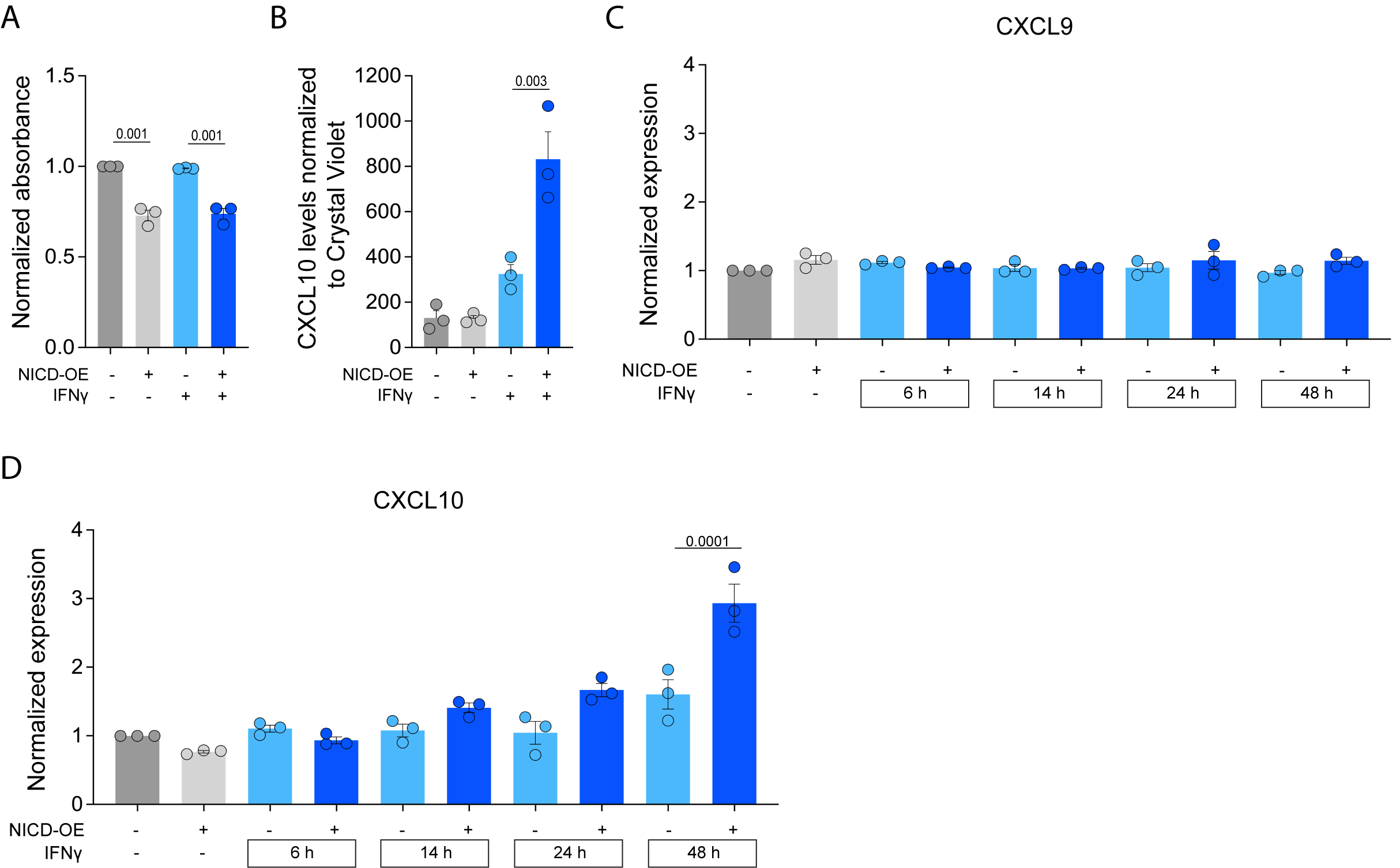

Supplement: Supplementary file 1 [file cimb-48-00547-s001.zip › Figure S4.tif]

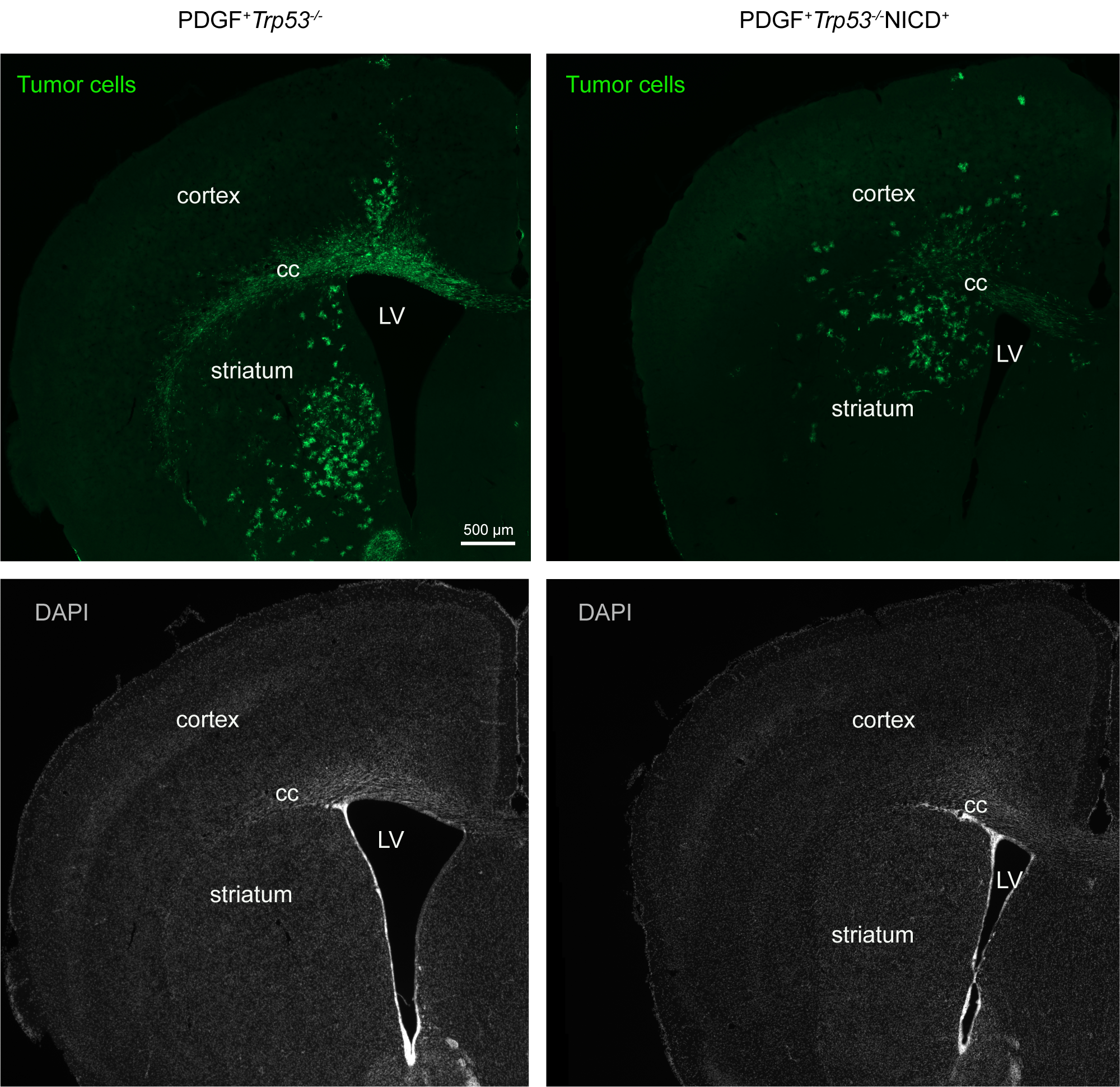

Supplement: Supplementary file 1 [file cimb-48-00547-s001.zip › Figure S5.tif]
